# Prediction of chemotherapy response in breast cancer patients at pre-treatment using second derivative texture of CT images and machine learning

**DOI:** 10.1016/j.tranon.2021.101183

**Published:** 2021-07-19

**Authors:** Hadi Moghadas-Dastjerdi, Shan-E-Tallat Hira Rahman, Lakshmanan Sannachi, Frances C. Wright, Sonal Gandhi, Maureen E. Trudeau, Ali Sadeghi-Naini, Gregory J. Czarnota

**Affiliations:** a Department of Medical Biophysics, University of Toronto, Toronto, ON, Canada; b Physical Sciences Platform, Sunnybrook Research Institute, Sunnybrook Health Sciences Center, Toronto, ON, Canada; c Department of Radiation Oncology, Odette Cancer Center, Sunnybrook Health Sciences Center, Toronto, ON, Canada; d Department of Radiation Oncology, University of Toronto, Toronto, ON, Canada; e Faculty of Engineering, University of Waterloo, Waterloo, ON, Canada; f Surgical Oncology, Odette Cancer Center, Sunnybrook Health Sciences Center, and Department of Surgery, University of Toronto, Toronto, ON, Canada; g Division of Medical Oncology, Odette Cancer Center, Sunnybrook Health Sciences Center, and Department of Medicine, University of Toronto, Toronto, ON, Canada; h Department of Electrical Engineering and Computer Science, Lassonde School of Engineering, York University, Toronto, ON, Canada

**Keywords:** Locally advanced breast cancer (LABC), Neoadjuvant chemotherapy (NAC), Quantitative computed tomography (qCT), Personalized medicine, Machine learning, Derivative textures

## Abstract

Although neoadjuvant chemotherapy (NAC) is a crucial component of treatment for locally advanced breast cancer (LABC), only about 70% of patients respond to it. Effective adjustment of NAC for individual patients can significantly improve survival rates of those resistant to standard regimens. Thus, the early prediction of NAC outcome is of great importance in facilitating a personalized paradigm for breast cancer therapeutics. In this study, quantitative computed tomography (qCT) parametric imaging in conjunction with machine learning techniques were investigated to predict LABC tumor response to NAC. Textural and second derivative textural (SDT) features of CT images of 72 patients diagnosed with LABC were analysed before the initiation of NAC to quantify intra-tumor heterogeneity. These quantitative features were processed through a correlation-based feature reduction followed by a sequential feature selection with a bootstrap 0.632+ area under the receiver operating characteristic (ROC) curve $\left(\text{AU}C_{0.632+}\right)$ criterion. The best feature subset consisted of a combination of one textural and three SDT features. Using these features, an AdaBoost decision tree could predict the patient response with a cross-validated $\text{AU}C_{0.632+}$ accuracy, sensitivity and specificity of 0.88, 85%, 88% and 75%, respectively. This study demonstrates, for the first time, that a combination of textural and SDT features of CT images can be used to predict breast cancer response NAC prior to the start of treatment which can potentially facilitate early therapy adjustments.

## Highlights

• Textural and second derivative textural features of CT images can be used in conjunction with machine learning models to predict breast cancer response to chemotherapy prior to the start of treatment.
• The proposed predictive model separates the patients at pre-treatment into two cohorts (responders/non-responders) with significantly different survival.
• The proposed methodology is a step forward towards the precision oncology paradigm for breast cancer patients.

## Introduction

According to recent cancer statistics, approximately 1 in 2 North Americans is expected to be diagnosed with cancer in their lifetime [1]. Among women, breast cancer is the most common cancer, and the second leading cause of cancer-related death worldwide. It accounts for nearly 30% of all new cancer diagnoses annually in women [1,2]. Locally advanced breast cancer (LABC) represents up to 20% of newly diagnosed breast cancer cases [3], [4], [5]. LABC includes stage III and a subdivision of stage IIB breast cancer, defined as bulky invasive tumors with a size of often greater than 5 cm which may include varying degrees of skin and/or chest wall involvement [5]. The 5-year survival rate for LABC is significantly lower than the rate for early-stage breast cancer [5,6]. The standard treatment for LABC includes neoadjuvant chemotherapy (NAC) followed by surgery and, if required, adjuvant radiation and/or hormonal therapy [7,8]. Although it has been shown that NAC outcome is correlated with long-term survival [9,10], this varies among different patients because of the factors such as clinical, pathologic and genomic characteristics of a patient, choice of chemotherapeutic agents and the frequency of treatment cycles. Although patients who have a pathologic complete response (pCR) demonstrate a significant better prognosis compared to other patients, about 70–80% of patients fail to achieve a pCR to standard NAC [4,11,12]. Predicting response to NAC before or early after the start of treatment can facilitate tailoring of chemotherapy for breast cancer patients in order to increase the likelihood of pCR. Presently, post-treatment anatomical imaging and/or histopathology on post-surgical specimens are standard methods to evaluate the tumor response to NAC. However, the window to adjust treatment is already closed at that time. Additionally, tumor size changes in response to chemotherapy often occur slowly to the extent that they are frequently not detectable on anatomical imaging even weeks to months after treatment initiation. Thus, effective biomarkers that can predict treatment response to standard NAC before to early after treatment initiation are critical in order to enhance treatment or make treatment modifications on an individual patient basis.

Various quantitative imaging techniques have been developed to evaluate therapy response in breast cancer patients early after the start of NAC. Previous studies have demonstrated the efficacy of positron emission tomography (PET) to monitor tumor response to chemotherapy early after treatment [13,14]. The main disadvantages of this technique include its high cost and a requirement of radionuclide contrast agent. In another study, dynamic contract-enhanced magnetic resonance imaging (DCE-MRI) has been utilized to predict the chemotherapy outcome for breast cancer patients [15]. Although it has shown promising results, MRI has not become a regular choice for NAC response prediction since it is expensive and is not always accessible. Diffuse optical imaging (DOI) has recently been investigated to monitor chemotherapy response in breast cancer [16,17]. DOI, however, is not commonly used in clinic as a standard methodology and its data acquisition for reconstruction of a volumetric image with reasonable resolution requires a lengthy time to completion [18]. Ultrasound (US) imaging has also been adapted in several studies to measure tumor response to chemotherapy [19], [20], [21]. Moreover, it has been shown that quantitative ultrasound (QUS) methods yields promising results in tissue characterization and tumor response monitoring [22], [23], [24], [25]. Nevertheless, US images cannot provide 3D volumetric information with the high quality readily achievable in MRI and CT images.

Recently, quantitative imaging techniques such as QUS and DOI have shown promise to predict the response to chemotherapy in LABC patients prior to the start of treatment [26,27]. Results from these studies have indicated that tumor aggressiveness and responsiveness to chemotherapy can be correlated to micro-structure and metabolic characteristics of tumor. The studies demonstrated that these characteristics can be revealed by QUS and DOI and utilized as predictive features of tumor response to treatment. In addition to those modalities, quantitative computed tomography (qCT) can potentially be used to quantify the micro-characteristics of a cancerous tumor. Usually, CT imaging is a part of the standard of care "work-up" for LABC patients being used to provide information regarding disease extent but also can reveal structural tumor information. The diagnostic information can be extracted more accurately from breast CT scans when contrast agents are used [28]. Contrast-enhanced CT (CE-CT) has shown promise to assess and monitor breast cancer response to NAC over the course of treatment [29]. Although the voxel size in CT images is multiple times larger than cellular dimensions, changes in tissue micro-structure could measurably alter the CT voxel intensities as a result of partial volume effects. In keeping with this, qCT techniques have successfully been applied for various tissue characterization applications including COPD diagnosis and staging [30,31], pulmonary fibrosis assessment [32], the measurement of mineral density [33] and bone mechanical properties [34], renal cell carcinoma differentiation [35,36], and differentiation of primary lung cancer and granulomatous nodules [37].

In [38] it was demonstrated that textural qCT biomarkers could be used for *a priori* prediction of tumor response to NAC in LABC patients. In that study, only the traditional first-derivative texture features of CT images were investigated. The promising results obtained motivate further investigations to explore higher-order features that can potentially improve predictive sensitivity and specificity. In keeping with this, a combination of textural and second derivative textural (SDT) qCT biomarkers have been investigated in this study for the *a priori* prediction of tumor response to NAC in LABC patients for the first time. A total of 72 quantitative features were extracted from the CE-CT images acquired prior to start of NAC for each patient. The features included 8 textural and 64 SDT features that were processed through feature reduction/selection. The selected features were utilized as qCT biomarkers in order to predict the treatment outcome of NAC for LABC patients using a machine learning approach. Results were validated based on standard histopathology on surgical specimens obtained many months later at post-treatment. It has been demonstrated that a personalized medicine approach, possibly facilitated by an early prediction of response to chemotherapy, can potentially enhance the survival and quality of life for cancer patients [39]. This study opens up a new chapter in early prediction of cancer response to treatment using quantitative imaging biomarkers.

## Materials and methods

### Study protocol and data acquisition

This study was conducted under regulations and guidelines in accordance with institutional research ethics board approval at Sunnybrook Health Sciences Center (SHSC), Toronto, ON, Canada. The study was open to all women aged between 18 and 85 years diagnosed with LABC and planned to undergo a full course of NAC followed by surgery. In keeping with this, 72 eligible patients diagnosed between 2009 and 2017 were enrolled in this prospective study, after providing informed consent. For all patients, a core needle biopsy was performed as part of institutional standard of care in order to confirm the cancer diagnosis. Additionally, the initial cellularity, histological subtype, and the hormone receptor status of the tumor were assessed using biopsy samples. Pre-treatment CE-CT images of the breast were acquired for all patients as part of the institutional standard of care. CT scans were performed with a multi-slice CT scanner (LightSpeed, GE Medical Systems, Chicago, United States). The scan parameters of tube voltage: 120 kV, X-ray tube current: 10–367 mA, slice thickness: 2.5 mm, pixel spacing: 0.8 × 0.8 mm, and slice size: 512 × 512 pixels were applied in helical mode. In order to measure tumor size and assess chest wall involvement, the patients underwent clinical MRI scans before and after the treatment as part of the institutional standard of care for LABC patients.

### Pathological evaluation of tumor response

After completing NAC, breast surgery was performed for all patients. About one third of patients went through a breast-conserving surgery (lumpectomy) while others underwent a mastectomy. Standard histopathology on the surgical specimens to assess the pathological response of tumor to NAC was carried out by board-certified pathologists as part of standard of care. The specimens were stained with hematoxylin and eosin (*H*&*E*) and prepared when possible on whole-mount 5″ × 7″ pathology slides which were digitized using a confocal scanner (TISSUEscope, Huron Technologies, Waterloo, Canada). Patients were categorized into two groups of responders (*R*) and non-responders (NR) using a modified response (MR) grading system which was based on RECIST [40] and histopathological criteria [41] as before [27]. The MR score was defined as follows: MR 1: no reduction in tumor size; MR 2: up to 30% reduction in tumor size; MR 3: 30% to 90% reduction in tumor size or a very low residual tumor cellularity determined histopathologically; MR 4: more than 90% reduction in tumor size; MR 5: no evident tumor and no malignant cells identifiable in sections from the site of the tumor; only vascular fibroelastotic stroma remaining, often containing macrophages; nevertheless, ductal carcinoma *in situ* may be present. The patients with a MR score of 1–2 (less than 30% reduction in tumor size) and 3–5 (more than 30% reduction in tumor size or with very low residual tumor cellularity) were determined as NR and *R*, respectively. In keeping with this, 56 and 16 patients were identified as responders and non-responders, respectively.

### Feature extraction and pre-processing

**Fig. 1:**
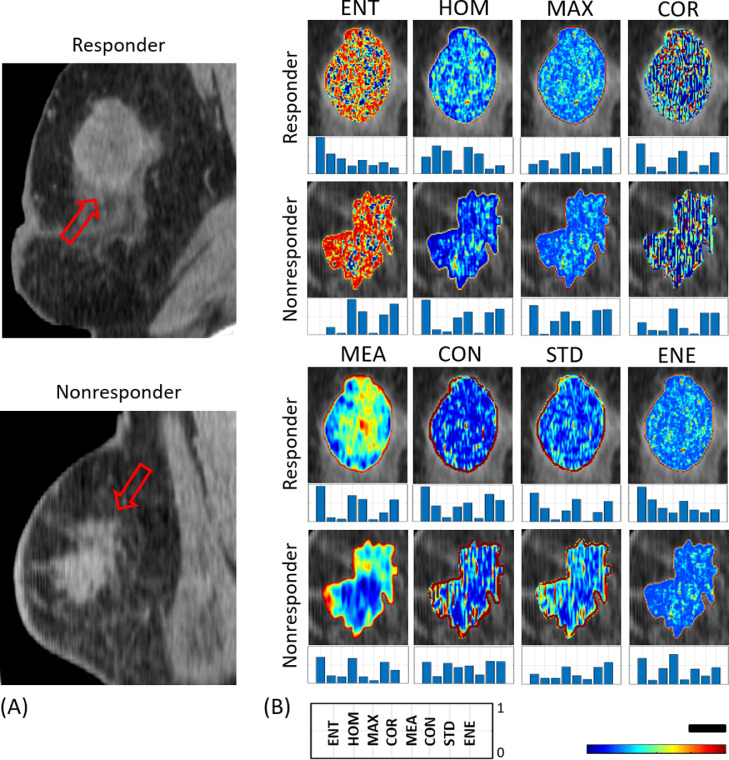
Representative CT images (A) with parametric map overlays (B) acquired for a responding and a non-responding patient. The parametric maps demonstrate entropy, homogeneity, maximum GLCM probability, correlation, GLCM mean, contrast, GLCM standard deviation and energy. The bar plots underneath the parametric maps show SDT feature values. These values normalized to a range of 0–1 for better demonstration. The box at the bottom of the figure illustrates the title of each bar. The color bar represents a scale of the range [1.5, 2.5] for ENT, [0, 0.8] for HOM, [-0.1, 0.8] for MAX, [-0.2, 0.7] for COR, [0, 50] for MEA, [0, 100] for CON, [-0.9, 8] for STD and [0.1, 0.8] for ENE. The scale bar represents 2 cm.

The regions of interest (ROI) were delineated manually on all slices of each 3D CT image to include the entire tumor. All the segmentation and imaging data analysis was performed by trained staff under the supervision of expert oncologists. Textural analysis was performed using a grey-level co-occurrence matrix (GLCM) methodology to generate textural parametric maps for each ROI and extract textural and SDT features [42]. The GLCM statistically assesses the angular and distance relationship between neighbouring pixels with different values and then characterizes the texture of an image by quantifying how frequently various pairs of pixel intensities occur in an image [42]. The voxel intensities in each CT image were initially quantized to 128 gray levels linearly. The parametric maps of textural features were then generated using a GLCM-based analysis on grey-scale CT images along with a sliding window approach. A three-pixel by three-pixel window was used to sweep the whole ROI with a step size of one pixel. The GLCMs were symmetrically computed over the one-pixel distance from each reference pixel at different directions with angles of 0°, 45°, 90° and 135° in each window. Eight textural features including entropy (ENT), contrast (CON), correlation (COR), maximum probability (MAX), mean (MEA), homogeneity (HOM), standard deviation (STD) and energy (ENE) were calculated for each GLCM. Corresponding equations for these textural features are described in the supplementary materials. The average value of each textural feature over the four GLCMs was assigned to the center pixel of the sliding window in the corresponding parametric map. The textural parametric maps were generated for all 2D slices to cover the entire tumor volume. The median number of slices for each tumor was 10. The mean-value of each parametric map was calculated to obtain the corresponding textural feature for that slice. The SDT features of each parametric map were also derived by applying a second GLCM-based textural analysis on the parametric map. Specifically, eight textural features of ENT, CON, COR, MAX, MEA, HOM, STD and ENE were derived as SDT features from each textural parametric map, e.g. ENT_CON represents the contrast feature of entropy parametric map (Fig. 1). As such, 8 textural and 64 (8 × 8) SDT features were derived from each parametric map. Since the area of tumor cross-section varies between different slices, a weighted averaging scheme based on the ROI area in each slice was utilized to compute the average of each feature over the entire tumor. Feature standardization was performed using the robust scaler method to make the numeric range of the features consistent [43]. The standardization was performed independently on each feature by computing the relevant statistics on the samples in the dataset to move the median to zero and scales the data according to the range between the 1st and 3rd quartiles. A Pearson correlation was utilized to analyze the inter-feature correlations followed by obtaining the coefficient of determination (*R^2^*) for each feature pair.

### Feature selection and response prediction

The features were analyzed independently in three different sets including only textural features, only SDT features, and all textural and SDT features. All the features in each set were first ranked using the minimal-redundancy-maximal-relevance (mRMR) criterion along with leave-one-patient-out (LOPO) cross validation [44]. Specifically, the features were ranked iteratively using the LOPO training data and the final ranking was based on the maximum number of occurrence in each position. For instance, the first feature was the feature which appeared the most in first position. The first eight features in the ranked list were retained for feature selection and the remaining features were removed from the feature list. The optimum feature subset was then selected by applying a sequential forward feature selection (SFS) scheme in conjunction with the iterative LOPO cross validation. The role of mRMR ranking in the feature selection process was only reducing the size of feature list and determining the first selected feature, i.e., the other feature rankings were not applied in the SFS process. Using the first feature in the mRMR ranking as the primary feature subset, the other features were iteratively added to the subset, while the performance of the subset in response prediction was evaluated using the $\text{AU}C_{0.632+}$ with the LOPO training data [45], [46], [47]. The $\text{AU}C_{0.632+}$ is designed to minimize the bias and variance when cross validation and/or bootstrapping is used for small datasets (details provided in supplementary materials). At the end of the process, the subset that yielded the highest $\text{AU}C_{0.632+}$ was selected as the optimum feature subset to train the classifiers.

In order to reduce the chance of a biased training due to the imbalance of the data, the dataset was balanced prior to the start of training procedure by oversampling the minority group to a double size using the SOMTE method [48,[49], [50], [51], [52], [53], [54], [55], [56]] and undersampling the majority group by taking *B* = 300 bootstrapped samples with the size of the oversampled minority group. The parameter configuration used for SMOTE included: random_state = 14, *k*_neighbors = 3, *m*_neighbors = 5. In order to achieve balanced and properly mixed training sets the oversampled minority subset were combined with each of the bootstrapped subsets from the majority group and then were randomly shuffled. These training sets were utilized to train adaptive boosting (AdaBoost) [57] classifiers with a decision tree (DT) model [58] as the weak learner (AdaBoost-DT). The hyperparameters of the AdaBoost DT classifiers were optimized based on a grid search as follows: maximum depth = 4, quantity of estimators = 150, learning rate = 0.1. Specifically, 300 classifiers were trained independently using the 300 balanced training sets. The performance of the outcome prediction models was evaluated using the LOPO cross-validation. The LOPO sample in each round was left out before performing the SMOTE oversampling and bootstrapping. As such, the test sample was isolated from the training set and the samples generated by SMOTE. The predicted label of each test sample was determined using a majority vote on the output of all the 300 classifiers (using a decision threshold of 50%). The training procedure and LOPO cross validation were repeated until all the samples were tested.

### Survival analysis

Patients were followed clinically up to 10 years after the start of treatment and their clinical data were recorded for recurrence-free survival analyses. The recurrence-free survival analysis highlights the efficacy of treatment by comparing the long-term outcome for different patient cohorts. It has been demonstrated that lack of appropriate tumor response can significantly negatively impact patient survival [9,10]. The ten-years recurrence-free survival curves were generated using a Kaplan-Meier (KM) analysis. The curves were generated for the two cohorts of responders and non-responders determined at post-treatment based on the clinical and histopathological criteria, and at pre-treatment based on cross-validated response prediction using the textural qCT features and a combination of textural and SDT qCT biomarkers. A log-rank test was used to assess for statistically significant differences between the survival curves of the two cohorts determined based on pathological criteria or qCT biomarkers. The curve separations were compared to assess which method can more effectively differentiate the patients in terms of long-term survival.

### Implementation of methods

The textural analysis and feature extraction methodologies were implemented in MATLAB (MathWorks Inc., MA, U.S.A). The feature selection, machine learning and survival analysis methods were implemented in Python using the Scikit-learn [59] and Lifelines [60] libraries, respectively. The codes were run on a Dell PC OptiPlex 3020 (Intel Core i5 3.30 GHz CPU, 8 GB RAM, Dell Inc, Round Rock, Tx, U.S.A) with a windows 7 (64 bit) operating system (Microsoft, Redmond, WA, U.S.A).

## Results

Table 1 summarizes the pathological and clinical characteristics of the patients. Seventy-two (*n* = 72) women participated in this study with an age in the range of 27–83 years with a mean and standard deviation of 52.7 ± 11.9 years. The majority of the patients were diagnosed with invasive ductal carcinoma (IDC) (93%), with only a few patients having invasive lobular carcinoma (ILC) (4%) and invasive metaplastic carcinoma (IMPC) (3%). Most of the tumors were characterized as grade II or III (98.6%). Additionally, tumors with positive estrogen (ER+) and progesterone (PR+) receptors were found in the majority of the patients (66% and 61% respectively), whereas 33% of the patients had tumors with positive Her2/Neu receptor (HER2+), and 22% of the patients had triple negative tumor (ER-, PR-, HER2-). On average, the post-treatment tumor size was 40% smaller than its initial size which was in the range of 1.3–12.8 cm. For NAC, 50% of the patients received doxorubicin (Adriamycin), cyclophosphamide followed by paclitaxel (Taxol) (AC-T), 42% received 5-fluorouracil, epirubicin, cyclophosphamide followed by docetaxel (FEC-D), 5% received doxorubicin, cyclophosphamide followed by docetaxel (Taxotere) (AC-D) and 3% received paclitaxel and cyclophosphamide (TC). Moreover, all patients with HER2+ tumors received monoclonal antibody tratuzumab (Herceptin) (TRA). The treatment regimen was not modified based on the imaging findings during this observational study.

**Table 1:** Clinical and pathological characteristics of patients.

Characteristics	Responders	Non-responders
	Mean ± Standard Deviation	
**Age**	52.1 ± 11.9 years	54.6 ± 11.7 years
**Initial Tumor Size**	5.7 ± 2.9 cm	5.4 ± 2.3 cm
**Histology**	Percentage (Count)	
Invasive Ductal Carcinoma	96% (54)	81% (13)
Invasive Lobular Carcinoma	2% (1)	13% (2)
Invasive Metaplastic Carcinoma	2% (1)	6% (1)
**Tumor Grade**	Percentage (Count)	
Grade I	2% (1)	0% (0)
Grade II	48% (27)	62% (10)
Grade III	50% (28)	38% (6)
**Molecular Features**	Percentage (Count)	
ER+	64%(36)	75% (12)
PR+	60% (34)	62% (10)
HER2+	41% (23)	6% (1)
ER- / PR- / HER2-	21% (12)	25% (4)
ER+ / PR+ / HER2	23% (13)	0 (0)
ER+ / PR+ / HER2-	36% (20)	62% (10)
ER- / PR- / HER2+	11% (6)	0 (0)
**Residual Tumor Size**	2.0 ± 2.9 cm	7.1 ± 4.6 cm
**Response**	Percentage (Count)	
Responding Patients	78% (56)	-
Non-responding Patients	-	22% (16)

Fig. 1 demonstrates representative CT images with parametric map overlays of eight GLCM textural features acquired for a responding and a non-responding patient prior to NAC initiation. The normalized values of SDT features are depicted in bar plots underneath the corresponding parametric maps. Different patterns of spatial variation could be observed in parametric maps associated with the responding and non-responding patients. The SDT values could reveal quantitatively the differences between the parametric maps of the responding and non-responding patients.

**Fig. 2:**
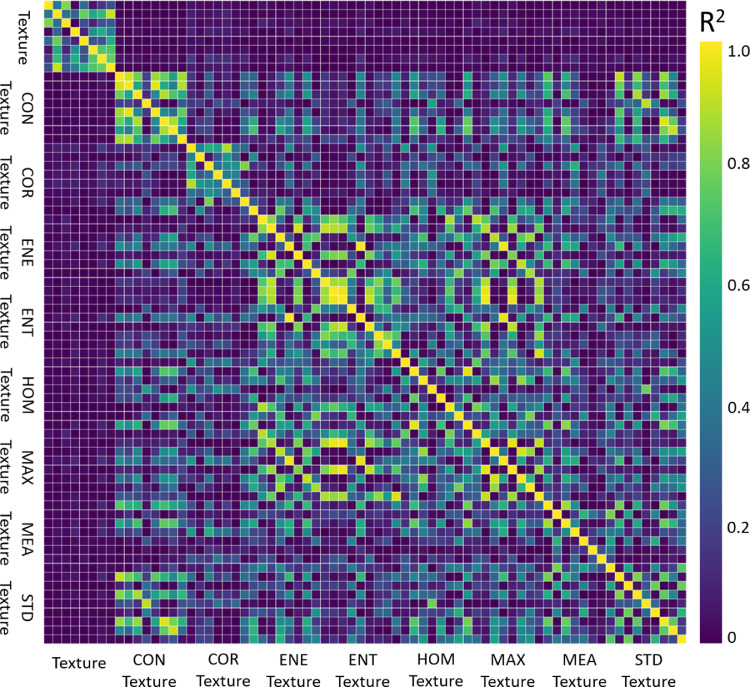
Heat map of the inter-feature correlations for the textural and SDT features. The values demonstrate the coefficient of determination (*R^2^*).

The inter-feature correlations are demonstrated in Fig. 2. This figure shows a heat map generated based on the coefficients of determination (*R^2^*) between all the feature pairs. The textural features demonstrated low levels of correlation with SDT features. This observation suggests that extracting second-pass textural features from the textural parametric maps can potentially reveal new information with minimal redundancy.

**Fig. 3:**
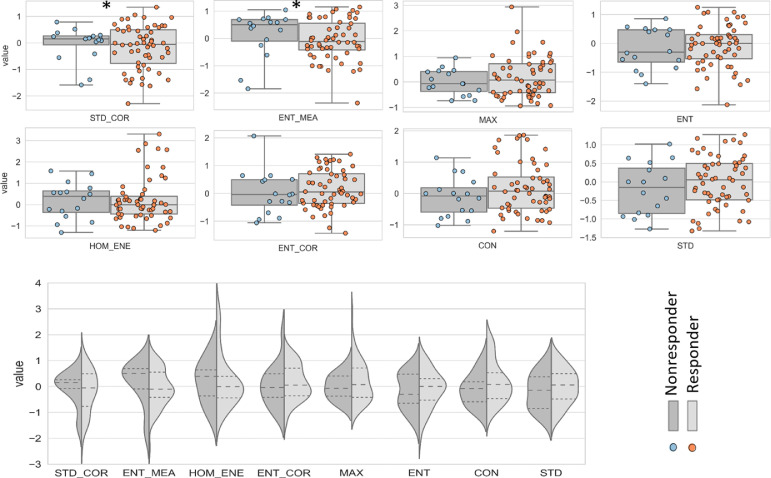
Statistical distribution of the selected feature values between the two groups of patients, i.e. responders and non-responders. The * in STD_COR and ENT_MEA figurers shows statistical significance (*p*-value < 0.05). In the bottom figure, the dash lines show the quartiles. All features were normalized according to a range between first and the third quartiles of their distribution.

Fig. 3 compares the statistical distribution of the selected feature values for the responding and non-responding patients. Although the features demonstrated a comparable range of values for the two patient cohorts, the mean values of ENT_MEA and HOM_ENE were notably different. Specifically, ENT_MEA were -0.066 and 0.155, and HOM_ENE were 0.255 and 0.189 for responding and non-responding patients, respectively. Additionally, in comparison to non-responding cohort, responding patients demonstrated a wider distribution in STD_COR and MAX features. A *t*-test (two-sided, α = 0.05) was used to compare the selected features in the two response cohorts. Results indicated that STD_COR and ENT_MEA were statistically significantly different between the two patient populations, with *p*-values of 0.030 and 0.037, respectively. However, the combination of the features provided the highest discriminatory power between the responding and non-responding patients.

The results of cross-validated response prediction are summarized in Table 2. Three different feature sets were investigated independently in which the optimum feature subsets consisted of textural features, the SDT features and a combination of the textural and SDT features (hybrid features). The optimum textural feature subset included ENT, MAX, CON and STD, whereas STD_COR, ENT_MEA, HOM_ENE and ENT_COR were selected as the optimum SDT feature subset. On the other hand, when the feature selection process was not restricted to select only from textural or SDT features, the optimum feature subset consisted of three SDT and one textural features, *i.e.* STD_COR, ENT_MEA, HOM_ENE and MAX. In terms of response prediction, the hybrid feature subset provided the most promising results with cross-validated scores of on $AUC_{0.632+}$= 87.7%, accuracy = 84.7%, specificity = 75%, sensitivity = 87.5%, precision = 92.5% and f-score = 89.9%.

**Table 2:** Performance of the outcome prediction models with textural, SDT, and hybrid features.

Features	$\text{AU}C_{0.632+}$ (%)	Accuracy (%)	AUC (%)	Specificity (%)	Sensitivity (%)	Precision (%)	F-Score (%)
ENT, MAX, CON, STD	74.3	77.8	69.2	62.5	82.1	88.5	85.2
STD_COR, ENT_MEA, HOM_ENE, ENT_COR	86.5	83.3	81.6	75.0	85.7	92.3	88.9
STD_COR, ENT_MEA, HOM_ENE, MAX	87.7	84.7	82.5	75.0	87.5	92.5	89.9

**Fig. 4:**
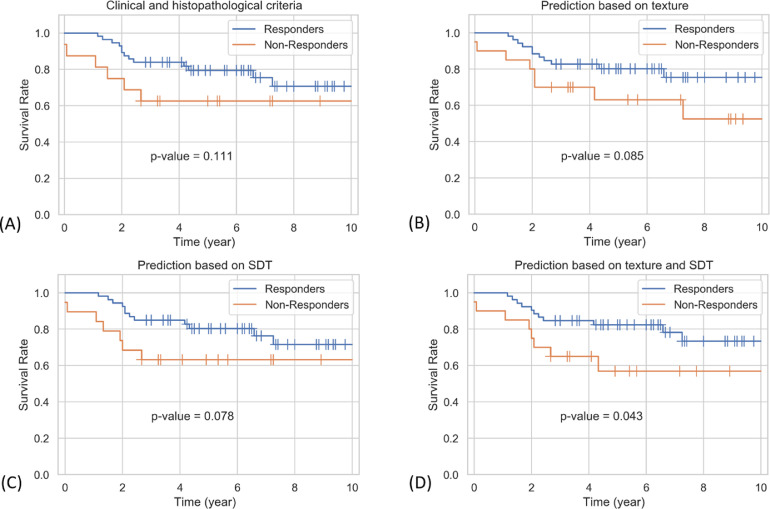
Ten-year recurrence-free survival curves for the responders and non-responders to NAC determined at: the end of treatment based on clinical and pathological criteria (A), and prior to the start of treatment based on prediction using the textural qCT biomarkers (B), SDT qCT biomarkers (C), and the hybrid textural and SDT qCT biomarkers (D).

Fig. 4 presents ten-year recurrence-free survival curves for the responding and non-responding patients determined based on the clinical and histopathological criteria at post-treatment, and outcome prediction at pre-treatment using the qCT biomarkers. In Fig. 4A patients were classified as responders or non-responder based on the clinical and pathological response. In Fig. 4B–D patients were classified based on the outcome prediction using the optimum qCT textural, SDT and hybrid (textural and SDT) biomarkers, respectively. The responding and non-responding patients demonstrated a ten-year survival rate of approximately 75% and 40–60%, respectively, in the three survival curves. Although the trends follow similar patterns in the curves generated based on the histopathological response and qCT prediction, as it can be seen in Fig. 4D the predictive model based on a combination of textural and SDT features could separate the patients into two cohorts with a more considerable difference in terms of survival. The survival curves of the responding and non-responding patients predicted based on the hybrid qCT biomarkers were in fact statistically significant with a *p*-values of 0.043.

## Discussion and conclusions

The results presented in this study demonstrated for the first time that a combination of SDT and textural qCT biomarkers could be used to predict LABC responses to NAC before the start of treatment with a high accuracy. Contrast-enhanced CT images were acquired from 72 breast cancer patients diagnosed with LABC prior to chemotherapy initiation. The patients were monitored during and followed up after the course of their neoadjuvant chemotherapy. The treatment responses were determined based on the standard clinical and histopathological methods after the completion of the course of NAC to validate the outcome predictions. Textural and SDT features were extracted using a GLCM method from the CT images and CT textural parametric maps, respectively. Seventy two (8 textural and 64 SDT) features were ranked using the mRMR algorithm. After removing highly correlated features, they were fed into a sequential forward feature selection technique based on $AUC_{0.632+}$ criterion to find the optimum feature subset. The optimum hybrid feature subset consisted of one textural (MAX) and three SDT (STD_COR, ENT_MEA, HOM_ENE) features. The performance of the response prediction model was evaluated using a LOPO cross validation. The AdaBoost-DT classifier using the optimum hybrid feature subset could predict the therapy response with a cross-validated accuracy, f-score and on $AUC_{0.632+}$ of 84.7%, 89.9% and 87.7%, respectively. The performance of the predictive models were similar in this study among the patients receiving different chemotherapy regimens, with the number of mispredictions proportional to the population size for the major regimens administrated (AC-T and FEC-D, administrated for 92% of the patients). Available options of therapy alteration/adjustments for patients with minimal response to a standard treatment may include modifying the regimen, dose and/or sequence of treatment options.

Since the spatial resolution of clinical CT images is relatively low, the details of cellular structures cannot be visualized in these images. Nevertheless, as a result of partial volume effect, variations in tissue micro-structure can still be partially detected in CT images in which each voxel intensity represents the weighted average of attenuation coefficients corresponding to all elements within the voxel [30]. On the other hand, it has been demonstrated that tumor aggressiveness and responsiveness to chemotherapy can be correlated to its micro-structural characteristics [27,[61], [62], [63]]. Consequently, the textural features quantifying spatial variations in the CT voxel intensities can be used to characterize underlying tumor tissue micro-structure. In keeping with this, the SDT qCT biomarkers indicated considerable differences between patients with responding versus non-responding tumors prior to start of chemotherapy, with two features (STD_COR and ENT_MEA) demonstrating statistical significance.

The number of responding and non-responding patients was not equal in this study. To address this issue, the dataset was balanced using a hybrid undersampling/oversampling method. The oversampling of minority and the undersampling of majority was obtained utilizing SMOTE and bootstrapping methods, respectively. Results indicated that the applied strategy improved the performance of the classifiers by preventing them from over-fitting toward the majority group. Specifically, the misclassification rate of the majority class reduced considerably (20% less misclassification rate for the majority class compared to the case of no undersampling/oversampling applied) while the misclassification rate of the minority class was not affected.

The effectiveness of textural analysis in breast cancer chemotherapy response prediction has been reported by multiple previous studies using different imaging modalities [17,26,27,38,[64], [65], [66]]. It has been demonstrated in [17] that DOI-based textural and mean-value parameters can quantify the functional and metabolic changes in LABC tumors in response to NAC. Moreover, in [64,67], researchers compared textural parameters, e.g. contrast, variance and entropy, in contrast-enhanced MR images prior to initiation of chemotherapy treatment and found significant differences between responding and non-responding patients. Chen *et al.* has demonstrated that after two cycles of treatment, the alternation of textural features in 18F-FDG PET images could reveal the signs of pathological complete response to NAC in both HER2- and HER2+ patients [65]. In another study, quantitative ultrasound textural features have shown promise to quantify micro-structural characteristics of tumors in the prediction of response to chemotherapy [27]. Meanwhile, recent studies have utilized the correlations between the anatomical and micro-structural information within US and CT to simulate US images from CT images [17,40]. These results in conjunction with the findings in studies that have used QUS to predict NAC outcome, could support the presented observations in this paper. Acquiring contrast-enhanced CT images is a standard part of pre-treatment disease evaluation for breast cancer patients in many cancer centers [40]. These available images can potentially be utilized to predict the likelihood of chemotherapy outcome by quantifying microstructural characteristics of tumor. The efficacy of using textural qCT biomarkers to predict response to NAC in LABC patients has shown promise in our previous study [38] and motivated the idea of using a combination of textural and SDT features for a more robust outcome prediction. Results indicated that the utilization of these combined qCT biomarkers could lead to an improvement in both the sensitivity and specificity of response prediction. Although detecting patients not responding to standard treatment is critical for a timely treatment adjustment, unnecessary modification of standard treatment can influence the quality of life and increase therapeutic side effects for responding patients. Consequently, an equal importance was assigned to both a high true positive rate as well as a low false positive rate to achieve a reasonable trade-off between sensitivity and specificity. The 10-year recurrence-free survival analysis demonstrated that hybrid textural and SDT qCT biomarkers acquired prior to the treatment initiation can better separate the patients in terms of long-term survival. A statistically significant difference was observed between the survival curves of the two patient cohorts identified by the prediction model using the hybrid textural and SDT qCT biomarkers (*p* < 0.05).

The principle here is to develop a methodology which can be used by physicians to modify chemotherapy treatment. If a patient is predicted with a sufficiently high accuracy to not respond to standard chemotherapy then an alternative regimen can be selected instead of having a patient undergo 4,5 months of a treatment that will not work. More efficacious regimens could be selected instead in such situations. CT Scanning is ideal in such a situation since it is already commonly used as part of patient care routinely thus requiring no additional capital resources. Comparison and fusion of the information from all modalities available for therapy response prediction at pre-treatment would be very interesting and facilitate development of multi-modal predictive models. However, usually very few imaging modalities are used for diagnosis and treatment planning of each patient as part of standard of care. Acquiring a prospective multi-modal dataset for this purpose would permit such investigations in future studies. A prospective multi-modal dataset if integrated with clinical, histopathological, and possibly genomic data of the patients would facilitate a thorough and rigorous investigation of various potential predictors of NAC response, along with comparative studies on the performance of single and hybrid therapy response biomarkers derived from imaging and non-imaging based information.

In conclusion, a new quantitative imaging method was presented in this study to predict LABC tumor response to chemotherapy prior to start of the treatment. In this technique, textural and SDT features of CT images were utilized as survival-linked qCT biomarkers of response to NAC. The results of this pilot study are promising, albeit obtained through cross-validation for feature selection and classifier evaluation due to the relatively small size of the dataset. Motivated by these results, a future large-cohort study with an independent test set is required to investigate the efficacy of the proposed biomarkers more rigorously and evaluate their generalizability. Acquiring data from a larger cohort of patients will also permit stratified analyses in terms of breast cancer sub-types and treatment regimens to explore further the effect of such factors in performance of the developed models. Additionally, availability of larger datasets would permit investigating other texture quantification methods, as well as advanced machine learning models with numerous parameters to be learned including the deep learning techniques for analyzing pre-treatment CT images for NAC response prediction.

## CRediT authorship contribution statement

**Hadi Moghadas-Dastjerdi:** Conceptualization, Methodology, Data curation, Software, Formal analysis, Writing – original draft. **Shan-E-Tallat Hira Rahman:** Data curation, Software. **Lakshmanan Sannachi:** Methodology, Data curation. **Frances C. Wright:** Resources. **Sonal Gandhi:** Resources. **Maureen E. Trudeau:** Resources. **Ali Sadeghi-Naini:** Conceptualization, Supervision, Methodology, Funding acquisition, Resources, Writing – review & editing. **Gregory J. Czarnota:** Conceptualization, Supervision, Methodology, Funding acquisition, Resources, Writing – review & editing.

## Declaration of Competing Interest

The authors declare no competing interests.

## Appendix Supplementary materials

Image, application 1
