# Supplementary material for: Prediction of chemotherapy response in breast cancer patients at pre-treatment using second derivative texture of CT images and machine learning
Source: Transl Oncol. 2021 Jul 19;14(10):101183. doi: 10.1016/j.tranon.2021.101183 (PMC8319580; doi:10.1016/j.tranon.2021.101183)
Supplement: Supplementary file 1 [file mmc1.pdf]

Supplementary Information

Prediction of Chemotherapy Response in Breast Cancer Patients at Pre-Treatment using Second Derivative Texture of CT Images and Machine Learning

Hadi Moghadas-Dastjerdi[1,2,3,4], Shan-E-Tallat Hira Rahman[2,5], Lakshmanan Sannachi[1,2,3,4], Frances C. Wright[6], Sonal Gandhi[7], Maureen E. Trudeau[7], Ali Sadeghi-Naini[1,2,3,8], Gregory J. Czarnota[1,2,3,4]

[1]Department of Medical Biophysics, University of Toronto, Toronto, ON, Canada.

[2]Physical Sciences Platform, Sunnybrook Research Institute, Sunnybrook Health Sciences Centre, Toronto, ON, Canada.

[3]Department of Radiation Oncology, Odette Cancer Centre, Sunnybrook Health Sciences Centre, Toronto, ON, Canada.

[4]Department of Radiation Oncology, University of Toronto, Toronto, ON, Canada.

[5]Faculty of Engineering, University of Waterloo, Waterloo, ON, Canada.

[6]Surgical Oncology, Odette Cancer Centre, Sunnybrook Health Sciences Centre, and Department of Surgery, University of Toronto, Toronto, On, Canada

[7]Division of Medical Oncology, Odette Cancer Centre, Sunnybrook Health Sciences Centre, and Department of Medicine, University of Toronto, Toronto, On, Canada

[8]Department of Electrical Engineering and Computer Science, Lassonde School of Engineering, York University, Toronto, ON, Canada.

Methods

Feature Extraction

The feature extraction pipeline was developed using the MATLAB libraries. Specifically, the texture analysis was performed using the Gray-level Co-Occurrence Matrix (GLCM) method implemented in MATLAB to quantify intra-and peri-tumor heterogeneities. The GLCM was presented to quantify statistically the angular relationship between the neighbouring pixels [1]. Symmetric GLCM matrices were created over the one-pixel distance from each reference pixel at four angular directions: 0°, 45°, 90°, and 135°. From the GLCM matrices, the GLCM features were derived including entropy (ENT), contrast (CON), correlation (COR), maximum probability (MAX), mean (MEA), homogeneity (HOM), standard deviation (STD) and energy (ENE):

$$Contrast = \sum_{|i-j|=0}^{N_g-1} |i-j|^2 \sum_{i=1}^{N_g} \sum_{j=1}^{N_g} p(i,j) \qquad (1)$$

$$Mean = \frac{\mu_i + \mu_j}{2} = \frac{\sum_{i=1}^{N_g} ip(i,j) + \sum_{j=1}^{N_g} jp(i,j)}{2} \qquad (2)$$

$$Standard\ Deviation = \sqrt{\frac{{\sigma_i}^2 + {\sigma_j}^2}{2}} = \sqrt{\frac{\sum_{i=1}^{N_g}\sum_{j=1}^{N_g}(i-\mu_i)^2 p(i,j) + \sum_{i=1}^{N_g}\sum_{j=1}^{N_g}\left(j-\mu_j\right)^2 p(i,j)}{2}} \qquad (3)$$

$$Energy = \sum_{i=1}^{N_g} \sum_{j=1}^{N_g} p(i,j)^2 \qquad (4)$$

$$Homogeneity = \sum_{i=1}^{N_g} \sum_{j=1}^{N_g} \frac{p(i,j)}{1+|i-j|} \qquad (5)$$

$$Entropy = -\sum_{i=1}^{N_g} \sum_{j=1}^{N_g} p(i,j)\ \log p(i,j) \qquad (6)$$

$$Correlation = \sum_{i=1}^{N_g} \sum_{j=1}^{N_g} \frac{(i-\mu_i)\left(j-\mu_j\right)p(i,j)}{\sigma_i \sigma_j} \qquad (7)$$

$$Max = \max_{i,j} p(i,j) \qquad (8)$$

Where the $p(i, j)$ is the probability of having neighboring pixels of intensities i and j in the image, and Ng denotes the number of gray levels (128). The μ and σ are the mean and standard deviation for row i or column j of the GLCM matrix. Textural features were subsequently averaged over the GLCMs obtained for different distances and angular directions. Among the extracted textural features, contrast quantifies local gray level variations in an image. Smoother image produces a lower contrast, while coarser image results in a higher contrast. Correlation represents linear correlation between neighboring pixels. Energy measures textural uniformity in an image. Homogeneity quantifies the incidence of pixel pairs of different intensities.

Classifier Evaluation

The accuracy, sensitivity, specificity, accuracy, precision, f-score, and $AUC_{0.632+}$ were obtained using the equations below:

$$Sensitivity = \frac{True\ Predicted\ Responders}{True\ Predicted\ Responders + False\ Predicted\ Nonresponders} \tag{9}$$

$$Specificity = \frac{True\ Predicted\ Nonresponders}{True\ Predicted\ Nonresponders + False\ Predicted\ Responders} \tag{10}$$

$$Accuracy = \frac{True\ Predicted\ Responders + True\ Predicted\ Nonresponders}{Totall\ Numbrer\ of\ patients} \tag{11}$$

$$Precision = \frac{True\ Predicted\ Responders}{True\ Predicted\ Responders + False\ Predicted\ Responders} \tag{12}$$

$$Recall = \frac{True\ Predicted\ Responders}{True\ Predicted\ Responders + False\ Predicted\ Nonresponders} \tag{13}$$

$$\mathrm{F-Score} = \frac{2 \times Persicion \times Recall}{presicion + Recall} \tag{14}$$

$$AUC_{0.632+} = \alpha_b AUC_b' + (1 - \alpha_b) AUC_a \tag{15}$$

$$AUC_b' = max\{0.5, AUC_b\} \tag{16}$$

$$\alpha_b = \frac{0.632}{1 - 0.368\, R_b} \tag{17}$$

$$R_b = \begin{cases} 1 & if\ AUC_b \leq 0.5 \\ \dfrac{AUC_a - AUC_b}{AUC_a - 0.5} & if\ AUC_a > AUC_b > 0.5 \\ 0 & otherwise \end{cases} \tag{18}$$

where AUC_a is the resubstitution AUC and AUC_b is the AUC of cross-validation [2]. A detailed description of the $AUC_{0.632+}$ method can be found in [2].

Reference

[1] R. M. Haralick, K. Shanmugam, and I. Dinstein, "Textural Features for Image Classification," *IEEE Trans. Syst. Man. Cybern.*, vol. SMC-3, no. 6, pp. 610–621, Nov. 1973.

[2] B. Sahiner, H.-P. Chan, and L. Hadjiiski, "Classifier performance prediction for computer-aided diagnosis using a limited dataset," *Med. Phys.*, vol. 35, no. 4, pp. 1559–1570, Mar. 2008.

[3] B. Efron and R. Tibshirani, "Improvements on Cross-Validation: The 632+ Bootstrap Method," *J. Am. Stat. Assoc.*, vol. 92, no. 438, pp. 548–560, Jun. 1997.
